# Experience of taking care of children exposed to HIV: a trajectory of expectations^1^

**DOI:** 10.1590/0104-1169.3607.2489

**Published:** 2014

**Authors:** Willyane de Andrade Alvarenga, Giselle Dupas

**Affiliations:** 2 Doctoral student, Escola de Enfermagem de Ribeirão Preto, Universidade de São Paulo, WHO Collaborating Centre for Nursing Research Development, Ribeirão Preto, SP, Brazil; 3 PhD, Associate Professor, Departamento de Enfermagem, Universidade Federal de São Carlos, São Carlos, SP, Brazil

**Keywords:** HIV, Infectious Disease Transmission, Vertical, Child Care, Nursing

## Abstract

**OBJECTIVE:**

to learn about the experience of caregivers/mothers providing care to infants exposed to HIV through vertical transmission.

**METHODS:**

this qualitative study used Symbolic Interactionism as the theoretical framework. A total of 39 caregivers of children exposed to HIV in follow-up at a specialized service were interviewed. Data were analyzed through inductive content analysis.

**RESULTS:**

four categories were identified that report on the lonely experience of handling the child's antiretroviral therapy, mainly due to a lack of information or incomplete information; being attentive to required care, such as the use of prophylaxis for pneumonia, vaccines, and other practices restricted to the mother-child interaction; the desire to omit the HIV out of fear of prejudice and fear of the disease, considering future prospects.

**CONCLUSION:**

the HIV and the threat this infection may affect the child cause apprehension and feelings such as fear, guilt and anxiety in the caregivers. Healthcare workers need to work together with mothers so they are able to cope with demands and distress. Only then will the treatment to avoid vertical transmission be efficient and will mother and child be supported during the process, despite apprehension with the outcome.

## Introduction

One of the diseases surrounded by stigma that most strongly affects families is the infection by the Human Immunodeficiency Virus (HIV). Healthcare workers play an essential role in implementing care and preventive measures among these families^(^ 1 ^)^. Approximately 35.3 million people live with the virus around the world^(^ 2 ^)^. In Brazil, this figure is approximately 718 thousand people. Since the identification of the first case in 1980 up to June 2013, a total of 686,479 cases have been reported, 64.9% of which among men and 35.1% in women^(^ 3 ^)^.

The infection among women is a cause of concern due to the transmission from mother to child. A total of 260 thousand new infections by HIV were reported among children with low and moderate income in 2012^(^ 2 ^)^. In Brazil, 93.6% of the individuals younger than 13 years old with HIV were contaminated by their mothers and this is the main infection route among children^(^ 3 ^)^.

A series of measures has been available in Brazil since 1996, which have the potential to reduce vertical transmission (VT) of HIV to less than 1%, namely: anti-HIV screening during prenatal care, the use of antiretroviral prophylaxis (ARP) during pregnancy and delivery, cesarean delivery for women with high or unknown viral load, replacement of breastfeeding by formula, the use of ARP in newborns plus medication to prevent pneumonia in the first months of life^(^ 4 ^)^.

Infection from mother to child presents high rates in Brazil despite universal access to ARP and care measures recommended by the Ministry of Health. Additional measures are required to effectively control the number of infants infected by HIV because studies report that the main factors accounting for VT include not testing for HIV during prenatal care^(^ 5 ^)^, poor information provided by healthcare workers^(^ 6 ^)^, and errors in the administration of ARP to the child^(^ 7 ^)^. Such evidence is part of the process of infant care and constitutes strong justification for the development of this study.

Therefore, nursing professionals are considered as having a key role in the care delivered to children, since they provide information and orientation concerning the therapy. The mother has many responsibilities concerning the child after hospital discharge, especially with regard to the ARP and chemoprophylaxis for pneumonia, among others^(^ 4 ^,^ 8 ^)^. Concomitantly, due to the initial lack of definition regarding the diagnosis^(^ 9 ^)^, for many months after birth, mothers experience fear that the child may be infected, have feelings concerning their HIV-positive status, and fear stigma since a Specialized Care Service (SAE) in HIV/AIDS is being used^(^ 7 ^)^.

Considering the importance of these issues and social interactions that surround the care provided to children exposed to HIV, these patients require that professionals are sensitive to their needs, to the needs of the caregiver/mother and those of the family, who provide care and experience a lack of definition regarding the child's diagnosis and also a feeling of being responsible for the VT. Hence, this study's aim was to understand the experience of caregivers/mothers with regard to the care provided to infants exposed to HIV due to vertical transmission.

## Method

This study adopted a qualitative, descriptive and exploratory method and employed Symbolic Interactionism as the theoretical framework, which sees the human being as a social being who interacts as a basic unit. From this perspective, individuals and society are created through social interaction and what happens in the present depends both on past experiences and relationships that take place in the present. Hence, if we want to understand the cause of human behavior, we need to focus on social interactions^(^ 10 ^)^.

Data were collected in a healthcare facility in the Northeast of Brazil, in a state university hospital founded in the 1970s that is kept with resources from the Brazilian Health System. It is considered a referral center for the care, prevention and treatment of individuals with HIV/AIDS and exposed children, both for those living in the state and in surrounding states. 

Selection criteria included: being a caregiver of a child up to 18 months old, not having a definitive diagnosis concerning HIV (infected or not infected/serum-reverted), and attending the follow-up in the service. This age group was selected because there is not a definitive diagnosis at this age due to the need to observe the serological evolution^(^ 4 ^)^.

A total of 36 caregivers and 29 children born from HIV seropositive mothers participated in this study: mothers (24), fathers (5), and others (7) such as grandmother, aunt, and great-grandmother. The number of participants was not previously established. Data collection ceased when no new reflections were presented and data became repetitive, a process called theoretical saturation^(^ 11 ^)^. Interviews were conducted from December 2012 to February 2013.

The adults responsible for taking care and accompanying the child to the service were approached in the waiting room and invited to talk in a private room the service had reserved for the interview. Those accompanied by other people who also took care of the child (e.g. father, grandmother, great-grandmother or aunt) were also invited to participate. They were asked to provide written consent and received clarification concerning the study objectives and strategy of data collection, and were also ensured of anonymity and confidentiality of their reports. Those who signed free and informed consent forms were included in the study.

The interview was collective when the caregiver was accompanied and individual when not accompanied. Interviews took 50 minutes on average and were conducted by the primary author. These interviews were conducted during a single meeting and the following questions were asked: "What is it like for you to take care of a child who was exposed to HIV?" and "What are the difficulties and facilities concerning the child's treatment?"

The interviews' audio was digitally recorded and transcribed verbatim. The content of interviews was grammatically corrected. The participants were identified by their kinship with the child (mother, father, aunt, grandmother, great-grandmother), followed by an ordinal number that showed the order of interviews.

Data were examined to verify what they revealed based on Qualitative Content Analysis, which consists of preparing, organizing and reporting results^(^ 12 ^)^. The gathering of data for content analysis, reading to obtain the meaning of the whole, and selecting units of analysis based on the study's objective and theoretical framework, permitted identifying the units of meanings, i.e. words, phrases or paragraphs related to the content and context. These first steps consisted of the preparation phase. In the organization phase, we opted for an inductive path, in which there was coding, the creation of categories and data were grouped within broader themes to reduce the number of categories. Hence, a general description of the phenomenon was developed through the generation of categories, abstracting them. The final phase consisted of reporting the results found in the previous phase through categories organized by a historical line of events concerning the phenomenon under study to provide the readers with meanings concerning the findings. 

The study received approval from the Institutional Board Committee at the Federal University of São Carlos (No. 112.500/2012). The current guidelines provided by the National Health Council Resolution 196 from 1996, in force at the time of the study's development and implementation, were complied with.

## Results

Most participants (23) were the mothers who had HIV and were the main caregivers. There was one foster mother and other people (grandmother, aunt and great-grandmother) who took care of the child due to the absence of biological mothers who either died or abandoned the child. When mother and child lived with the child's father or grandfather, these also took care of the child.

With regard to the age of the main caregivers (28), half were between 20 and 30 years old and the other half were older than 30 years of age. In terms of education, most participants had attended elementary and/or middle school (12), complete or incomplete high school (12), followed by those who attended some college or had a bachelor's degree (3), or were illiterate.

Regarding the discovery of HIV infection, there were mothers who found out before pregnancy (8), during pregnancy (14), during labor (2), and after the child's birth (4). Most (25) attended prenatal care and used ARP during pregnancy (24).

Of the 29 children (one case of twins) exposed to HIV, most were males (20), aged one to six months old (17). With regard to vertical exposure to HIV within the family, most reported it was the first case of exposure (22). Most children had siblings (17) and most fathers had HIV (13); five children had seronegative fathers and the serology of the fathers of ten children was not known or confirmed. Most children lived in the same city where the treatment/follow-up was provided but there were children living in other cities, up to 588 km away from the healthcare service. 

Four categories emerged from the content analysis process: *solitary experience in handling the antiretroviral therapy; paying attention to the care provided to the child; wishing to omit the HIV; and looking to the future and fearing the disease.*

## Solitary experience in handling the antiretroviral therapy

When ARP with zidovudine (AZT) was initiated after the child's birth in the maternity ward, it was administered by the nursing staff or this responsibility was transferred to the child's caregiver, either the mother or her companion. There were different schedules for the AZT administration and the hospital expected that the mother would bring the medication to be given to the child. This medication is provided by the specialized service (SAE) where the mother already attended her follow-up.

She [nurse] asked me whether I had brought the medication [AZT], I said no and they sent to get the medication at my place [...]. I didn't see them administer the first dose but the others I'd give to the baby myself. They would hand me the syringe with the dose and that's it (Mother 25).

The nurse would bring the medication and tell me to give it [...]. They wouldn't administer it! (Grandmother 16).

In this context, there were mothers who reported not being aware when the first dose of AZT was administered. Some were discharged from the hospital without knowing the name of the medication or its specificities. The caregivers administered the medication at home the same way they had witnessed at the hospital or the way they were oriented to. There were children who did not receive the medication to continue the therapy at home or initiated the therapy only at home. Some reported the supply of wrong or incomplete information, especially with regard to the date the therapy should be interrupted, so that some children received the medication for periods longer or shorter than the recommended.

They told me to do the same way they did at the maternity, every 8 hours [...]. She used it for 2 months, almost 3, I gave it until the bottle went dry because they told that I should give it to her up to the end (Mother 16).

He used AZT only at the maternity. The only thing they gave me at discharge was a prescription so I'd get the formula. [...] I didn't get medication or orientation. I called the service [SAE], but they told me that it was useless because more than 7 days had passed and there was no use in taking the medicine [...] (Mother 20).

The caregivers valued the medication even though they were unaware of its purpose. They reported being committed and concerned with complying with the therapy and some changed the family's routine to comply with the schedule and dedicated themselves so they would not forget administering the medication. In terms of believing in the medication, some caregivers believed that it was essential for the child to avoid infection by HIV or its manifestation.

They gave me AZT and told me that she should take it for a month, every 12 hours and she couldn't miss it not even for a day but they didn't tell me why. I didn't know what was that, had never heard of it. Nevertheless, I never stopped giving it and I thought: this medication is very important because I don't know what he has. I was afraid of not giving it and then after a while he'd be harmed and it would be my fault (Mother 26).

## Paying attention to the care provided to the child

This category gathered other care actions performed by the mother, such as administering sulfametoxazol associated with trimetoprima (SMZ-TMP). According to the mothers, this prevented pneumonia, while others did not even know what it was for. The participants reported that the children's vaccines were updated. Not all the participants reported the vaccines' side effects but when they did, side effects were not associated with HIV exposure. In general, the mothers did not report that the children presented any reaction to the medications, but only a difficulty in administering it because children regurgitate the medication, besides anemia associated with AZT. Some were not prepared for when the child regurgitates so they would become anxious and in doubt whether they should offer a second dose of medication.

After the AZT, she took sulfametoxazol [...]. Vaccination is updated. Of special vaccines, she took the inactivated polio [...] She has a temperature after another vaccine [...] She didn't have any side effects with these medications, sometimes she'd put it out and I'd give her again [...] I'd get pretty nervous (Mother 27).

He got anemia with this medication (AZT) he was taking, but the doctor said it was normal (Mother 11).

Mothers with HIV preventively established barriers in the care provided to the child or in their interactions living with the child in the same house. Blood was seen as the main transmission route; however, wounds, menstruation, kisses and sweat were reported as other routes. Even with guidance, the mothers were afraid of transmitting the virus. Even though the mothers had HIV, the children were considered healthy. HIV was a problem related only to the parents and, even when the children became sick, other elements were held accountable, such as meteorological variables like temperature, relative air humidity and precipitation, or associated with diseases prevalent during childhood.

[...] I'm afraid that he eats in the same plate as mine [...], I try not to sleep with him or put him in my bed and if I'm menstruated or with any injury I don't even get him on my lap [...] If I see blood, I think: this blood is contaminated, I don't kiss him, [...] If I could I wouldn't even touch him. I love him but I think he'll be safer if he stays away from me (Mother 20).

His health condition is good [...]. He had the flu some time ago. I think it was because of the weather, there was a lot of dust where I lived (Mother 9).

## Wishing to omit the HIV

HIV-related stigma, fear of prejudice and discrimination by others make mothers hide the HIV infection, both their own diagnosis and the exposure of their children. This fear is due to a previous experience in which she witnessed prejudice toward other people or to self-prejudice. She fears the child is rejected and, for this reason, the number of people aware of her serology and condition is restricted.

Our greatest concern is discrimination. Who knows it is only the closest family, father, mother and grandparents, the physicians who take care of me and nobody else. I see people talking about other people and I'm afraid to have to deal with it (Mother 3).

I fear for my daughter, that people will talk about what she has, for this reason I don't mention it to everyone. I'm afraid she suffers with prejudice (Mother 16).

When asked by others why the child is not breastfed, type of delivery, medications or regarding regular pediatric consultations, the mother provides an explanation other than HIV out of fear of prejudice. It caused suffering because mothers sometimes want to "vent" but are afraid their condition will not be accepted.

People would ask a thousand questions like why he'd not breastfeed. It was the worst part. I'd say I had anemia, that I didn't have milk [...]. I wouldn't tell the truth because not everyone accepts it (Mother 8).

People in my city ask questions, why he doesn't breastfeed? Why I have to go to the hospital? I say I'll take him to the pediatrician, because there is not a pediatrician there [city] and about breastfeeding, I say he didn't want to (Mother 2).

The fact that mothers have to attend a specialized service makes them feel exposed, fearing people's judgment, and that they will discover their condition. Being in the waiting room caused them discomfort because they feared being seen there, something they considered terrible. Whenever possible, they would deny the name of the hospital that hosted the specialized service and preferred general hospitals to attend consultations to keep the diagnosis "masked" or hidden. The caregiver feared that, when taking the child to other services such a Family Health Units (FHU), he would have to reveal his condition or that of the child.

We don't want them (FHU professionals) to know that we have it and it's even worse if it is where we live. Even though we know the professionals will not mention it, we think it may happen. We try to see whether she could have her follow-up at the maternity ward, but no, only here. It draws attention coming to a hospital of contagious diseases [SAE] and we wanted to hide it! (Father 10).

## Looking to the future and fearing the disease

The path up to the disclosure of the diagnosis was experienced with much anxiety, sadness, fear and doubt. The mother experienced uncertainty about the diagnosis with affliction and asked herself what the result would be and how it would be if it were different from what she expected. She awaited the result as a condition that would provide her with some relief but, up to the confirmation, she experienced mixed feelings, both positive and negative feelings. Strong influence of spirituality and faith in God, self-control and coping with the feeling of powerlessness were perceived. Faith and hope the child would be found negative prevailed.

I ask God every day that he doesn't have the disease. I have great expectations and this is what I expect. I see a good future for him, without the disease [...] what I expect most is that the doctor gives me a word of comfort, relief (Mother 12).

It is very sad for the caregiver to imagine the future of the child with HIV, whether because of all the struggle for it not happen or because someone in the family has suffered or died because of the disease. The expectation is to have a child free from HIV and the healthcare professional is seen as a hope for such a result. Seeing seropositive children in follow-up in the specialized service shows the possibility of their children to be in the same situation, which caused fear and sadness and, at the same time they acquired strength to accept the child and carry on with care in case the result was positive. 

If it is positive I'll get sad because I did everything I could. I believe he doesn't have it [...] I wonder: does he? I want to clarify this doubt. When he does the exam and it's positive, it'll be difficult to face it (Mother 28).

I always had faith, an expectation that everything will be ok. I think that he might have the virus, but at the same time I deny this thought and say to myself: even if he has it, there's treatment. I'll take care of him all the same (Aunt 19).

Because the caregivers have HIV, they fear death and consequently fear not being able to see their children grow and experience their rites of passage. The mother wonders who will take care of her child in her absence and, to avoid this outcome, she focuses on her treatment and healthy habits to achieve longevity.

I ask God for me to live longer and be able to take care of my children. I see that, when I have the certainty that he's not infected, I'll be happier and will try to take better care of myself and see they will become capable to live without me (Mother 22).

## Discussion

We sought to identify the experience of caregivers using the fundamentals of Symbolic Interactionism and the results revealed that their experiences are full of expectation concerning the children's diagnoses. This is so because the individual is a thinking being, active in the relationship with the environment. The meaning of things is the result of social interaction in the course of life, even though actions take place in the present, the interactions of the past interfere in present behavior and the future, which includes expectations, is also considered^(^ 10 ^)^.

Another Brazilian study reports similar results with regard to the higher number of male children being exposed to HIV and the number of siblings^(^ 8 ^)^. Women with HIV have had the highest number of pregnancies^(^ 13 ^)^ and the reason may be that prophylaxis to avoid VT is safe, which encourages them to have a new pregnancy^(^ 7 ^)^.

ARP should be initiated while the child is in the maternity ward, immediately after birth, and the mother or caregiver should receive the medications at hospital discharge and be able to continue the therapy at home^(^ 4 ^)^. In Brazil, AZT oral solution should be given to newborns exposed to seropositive mothers who received medication during pregnancy, preferably in the first four hours of life, in dosages that correspond to the newborn's number of gestational weeks, every 12 hours for four weeks. Newborns of infected mothers who did not receive APR during pregnancy should have AZT plus Neviparine, even those who received injectable AZT at the time of delivery^(^ 4 ^)^.

One study conducted in 30 Brazilian maternity wards assessed the degree of implementation of the Brazilian Program to Control the Vertical Transmission of HIV^(^ 5 ^)^, and another study that was conducted with mothers and children infected through VT^(^ 6 ^)^ reported weaknesses in the implementation of these measures by healthcare services, which corroborate this study's findings, especially with regard to lack of access to oral AZT. These weaknesses are due to a lack of organization, administration and assessment of healthcare services^(^ 5 ^-^ 6 ^)^. These failures may partially be related to the healthcare professionals^(^ 14 ^)^.

Mistakes may compromise the therapy's success. Among the main errors that take place at the patients' homes are: the administration of the AZT syrup beyond the recommended time and in inappropriate dosages, increasing the risk of side effects. According to a cross-sectional study addressing the ability of mothers to take care of children exposed to HIV in Brazil, the reason for the inappropriate administration of the medication is the lack of orientation at the maternity^(^ 8 ^)^, that is, low quality information is provided by the healthcare professionals. As stated by another study conducted with mothers of children younger than six months of age and exposed to HIV/AIDS, these are some of the main factors accounting for the current levels of VT in Brazil^(^ 7 ^)^.

One study reports that mothers are insecure and have difficulties administering the oral medication because the children do not completely swallow the AZT syrup^(^ 15 ^)^. With regard to AZT side effects, the mothers reported anemia, which was considered normal. Another medication that these children receive during the treatment is chemoprophylaxis for *Pneumocystis jiroveci *pneumonia (SMZ-TMP)*,* from four to six weeks of life until they complete one year old or until the possibility of HIV infection is completely cleared^(^ 4 ^)^. The reason is that pneumonia is the most frequent opportunist infection in the first year of life among children with HIV^(^ 16 ^)^.

The mothers believe in the treatment efficiency and that, when properly administered, the child will be healthy and free from HIV^(^ 7 ^)^. The mother or another caregiver is responsible for postnatal care and should be oriented to properly perform the therapy at home^(^ 17 ^)^. It has been a challenge because non-adherence to the treatment may cause the child to become infected by HIV, pneumonia or other comorbidities^(^ 16 ^)^. The mothers were committed to comply with the recommendations because they were afraid of transmitting HIV to their children. One qualitative study, also using Symbolic Interactionism as the theoretical framework, reports there is a desire and hope the child will be seronegative and achieving this goal is related to interactions between mother and child, mother and disease and mother and God^(^ 18 ^)^.

A scale was applied to 60 mothers to assess their ability to care for children exposed to HIV and found that they have moderate to high ability to administer AZT, with no significant association with education, age or income^(^ 17 ^)^. With regard to the immunization scheme, the mothers addressed in this study reported their children's vaccines were updates, different from other studies conducted in the Northeast of Brazil, reporting non-adherence to vaccines or inappropriate use of vaccines among children exposed to HIV^(^ 8 ^,^ 13 ^)^.

Having HIV or having a child at risk involves aspects that go beyond adherence to preventive measures. Caregivers have important roles and need to be supported in these roles^(^ 19 ^)^. Professional and family support is important for mothers who face the virus, stigma, and have the responsibility to care for these children^(^ 15 ^,^ 20 ^)^. Hiding their own diagnosis and the condition of their children is an option to avoid stigma, which limits support and prevents healthcare professionals from developing effective interventions. Mother and child avoid seeking healthcare services and social support and, therefore, become isolated, avoiding proximity to other people and sharing their experience with the disease out of fear that it may cause even more suffering^(^ 21 ^)^. The omission of their serological status is justified by the fear of judgment and stigma^(^ 22 ^)^.

The attitude of hiding their own diagnosis and the child's condition may harm the child's care and leave the parents vulnerable. This behavior, according to Symbolic Interactionism, derives from the internalization of the social process in which the individual acquires the ability to guide his/her actions, considering consequences foreseen for alternative lines of actions^(^ 10 ^)^. Therefore, the best option for parents is omission, because they fear the consequences of disclosing the diagnosis. These consequences involve meanings that are handled and modified through interpretative processes. Based on these processes, the individual directs his/her behavior or manipulates the situation^(^ 10 ^)^.

The way mothers interact with the disease interferes in her interaction with the child. The mother sees the disease as a threat in her everyday life while taking care of the child and in her contact with the child because she fears transmitting the disease to the child through skin wounds or casual contact^(^ 15 ^,^ 23 ^)^. The HIV prevents mothers from performing some everyday activities with their children^(^ 24 ^)^, as they see them as fragile and overprotect them, fearing that they will become ill^(^ 20 ^)^, even though they consider the children to be healthy^(^ 15 ^)^.

Their main concern is the child's definitive diagnosis. Their reaction when receiving the result of the HIV infection is related to the meaning assigned to a life with HIV. This meaning makes caregivers fear a positive diagnosis for their children. This is so, because human beings act in accordance with meanings and society is key for this because the meanings of things derive from the interaction with others^(^ 10 ^)^. The stigma of the disease and the importance of having close social relationships make the diagnosis to be feared, because it interferes at all levels of interaction, well being and success^(^ 21 ^)^.

Expectation with regard to the result is experienced with anxiety and apprehension. While certainty is not possible, the mothers resort to positive thinking and God, through faith, asking for the child not to become infected^(^ 18 ^,^ 20 ^)^. These interactions established with themselves and God help these mothers to cope with the situation.

## Conclusion

This study's objective was achieved. The presence of HIV and the threat of having a child infected cause apprehension and other feelings, such as fear, guilt, and anxiety among caregivers. The stigma of the disease, associated with lack of orientation, make mothers establish barriers in the interactions with their children, limiting their role and seeing themselves as a hazard, in the belief that they can transmit the virus to the child.

The caregivers are committed to fulfill the child's treatment even when not aware of the treatment's purpose. Mothers are also responsible for providing care and have to handle the ARP by themselves. This loneliness is already experienced at the maternity, where they have to assume this care procedure. Hence, it is at home and within their network of relationships that the caregiver omits the presence of the virus and deals with fears and expectations, waiting for the result of the child's diagnosis with hope and wondering about the future. 

The caregivers reported need for support, guidance and respect during the implementation of the child's treatment. Failures in the treatment and lack of professional support on the part of the healthcare services show inefficiency in complying with measures to control HIV VT. Note that these events took place in a particular context of healthcare delivery and results should not be generalized. 

Nonetheless, these results can collaborate to improvements by grounding training and professional practice with regard to what guidance caregivers demand. Healthcare workers, especially nursing professionals, being closer to patients, should work together with mothers, since the pregnancy, to comply with recommendations and cope with demands and distress.

Other studies addressing care provided at home, how to handle ARP, prejudice associated with HIV, communication between family and health staff, are necessary to gain a better understanding of the experience of caregivers taking care of children exposed to HIV. Only then will the treatment to avoid VT be efficient and will caregivers, despite fearing the result, experience this process better, with support and without prejudice.
